# Synergistic effect of nitrate-doped TiO_2_ aerosols on the fast photochemical oxidation of formaldehyde

**DOI:** 10.1038/s41598-017-01396-x

**Published:** 2017-04-25

**Authors:** Jing Shang, Wei Wei Xu, Chun Xiang Ye, Christian George, Tong Zhu

**Affiliations:** 1 State Key Joint Laboratory of Environmental Simulation and Pollution Control, College of Environmental Sciences and Engineering, Peking University, Beijing, 100871 People’s Republic of China; 2 Université Lyon 1, CNRS, UMR 5256, IRCELYON, Institut de recherches sur la catalyse et l’environnement de Lyon, 2 avenue Albert Einstein, F-69626 Villeurbanne, France

## Abstract

The uptake of formaldehyde (HCHO) on mineral dust affects its budget as well as particle properties, yet the process has not yet been fully investigate. Here, TiO_2_ and nitrate-doped TiO_2_ aerosols were used as proxies for mineral dust, and the uptake of HCHO was explored in a chamber under both dark and illuminated conditions. The uptake loss of HCHO on UV-illuminated aerosols is 2–9 times faster than its gaseous photolysis in our experimental system. The uptake coefficient in the range of 0.43–1.68 × 10^−7^ is 1–2 orders of magnitude higher than previous reports on model mineral dust particles. The reaction rate exhibits a Langmuir-Hinshelwood-type dependence on nitrate content and relative humidity, suggesting the competitive role of nitrate salts, water vapor and HCHO on the TiO_2_ surface. The reaction produces carbon dioxide as the main product and gaseous formic acid as an important intermediate. The hydroxyl radical produced on illuminated TiO_2_ primarily drives the fast oxidation of HCHO. The nitrate radical arising from the TiO_2_-catalyzed photoreaction of nitrate synergistically promotes the oxidation process. This study suggests a novel oxidation route for HCHO in the atmosphere, taking into account high abundance of both mineral dust and anthropogenic TiO_2_ aerosols.

## Introduction

Formaldehyde (HCHO) is the most abundant carbonyl compound in the atmosphere; its presence affects both the radical budget and secondary aerosol formation^1, 2^. For instance, photolysis of HCHO is an important source of HO_x_ (=OH radical +HO_2_ radical), which plays a critical role in the photochemical reactions in the troposphere^3^. Meanwhile, oxidation of HCHO produces formic acid, which increases the acidity of aerosol particles via gas-particle partitioning, promoting the formation of secondary organic aerosols (SOAs)^4, 5^. The oxidation of long-chain organic compounds, such as isoprene, is the major chemical source of HCHO in the low troposphere, while the oxidation of methane is the major chemical source in remote areas^6–8^. Although the chemical source dominates, direct emissions from combustion and vegetation are not negligible^9^. In addition, emission of HCHO from processes in the snow pack greatly affects the emission budget in snow-covered areas^10^. The sink of HCHO includes deposition, oxidative decay by hydroxyl radicals (·OH) and most importantly its photolysis in the gas phase^6–8^. The atmospheric lifetime of HCHO against its chemical decay is approximately one day, while its photolysis lifetime around noon is as short as 1–2 hours. Despite the detailed knowledge on the HCHO budget, a substantial discrepancy between field observations and model outputs has been found, both for box and multi-dimensional models^6–8^. The discrepancy suggests, in fact, an incomplete understanding of the budget of HCHO, such as the heterogeneous sink of HCHO on aerosols in regions of heavy aerosol loading^8, 11–14^.

The uptake and following oxidation or oligomerization of HCHO on aerosols has been explored in the laboratory as a potential sink of HCHO^11–13^. The uptake coefficient of HCHO on various aerosol components, including sulfuric acid, organic matters, and mineral dust, has been measured^11, 12, 15–17^. Generally, previous evaluations based on the laboratory measurements suggest that the heterogeneous sink of HCHO can be neglected^11, 12, 16, 18^. However, we argue here that previous studies have not fully explored the photo-enhancement effect of mineral dust (e.g., TiO_2_), nor possible synergistic effects of other aerosol constituents, such as nitrate, under environmentally relevant conditions^11, 12, 16, 18, 19^.

TiO_2_ is a minor but still an important component of mineral dust with a mass fraction in the range from 0.1–10 wt.%^20^. The injection of dust into the atmosphere is ~1,000–3,000 Tg per year^21, 22^. In addition, TiO_2_ is now increasingly used as an environmental self-cleaning and depolluting coating on the outer layer of buildings, glasses, airport roofs, etc. due to its photocatalytic properties^23–25^. *Bang and Murr* identified a TiO_2_ particle with a size of about 50 nm in atmospheric particulate matter in El Paso, Texas, with its source suspected to be anthropogenic^26^. Many laboratory studies have been conducted to explore the photocatalytic features of TiO_2_ in heterogeneous reactions. The results show that UV-illuminated pure TiO_2_ or mineral dust greatly enhanced the uptake of O_3_, NO_x_ (NO + NO_2_), SO_2_, volatile organic compounds (VOCs), compared to that under dark conditions^27–36^. Under a typical flux of 10^3^–10^4^ photons·cm^−2^ · nm^−1^ between 300–390 nm in the solar radiation^37^, excited TiO_2_ is theoretically able to generate photoactive species that drive rapid redox reactions of HCHO on mineral dust aerosols and photocatalytic anthropogenic surfaces.

Here, the reactions of HCHO on TiO_2_ and nitrate-doped TiO_2_ aerosols were investigated in a 400 L environmental aerosol chamber under 8–80% RH. The uptake of HCHO and formation of products were monitored as the chamber was illuminated under environment-relevant UV radiation in the region of 300–420 nm.

## Results

Below we demonstrate that the oxidation of HCHO is significantly photo-enhanced, with a synergistic effect from the photoreaction of nitrate on the aerosol. The uptake loss of HCHO in our experimental system is even faster compared to its gas-phase photolysis, inferring a novel sink of HCHO of potential atmospheric significance.

### Fast Photochemical Oxidation of HCHO

**Figure 1:**
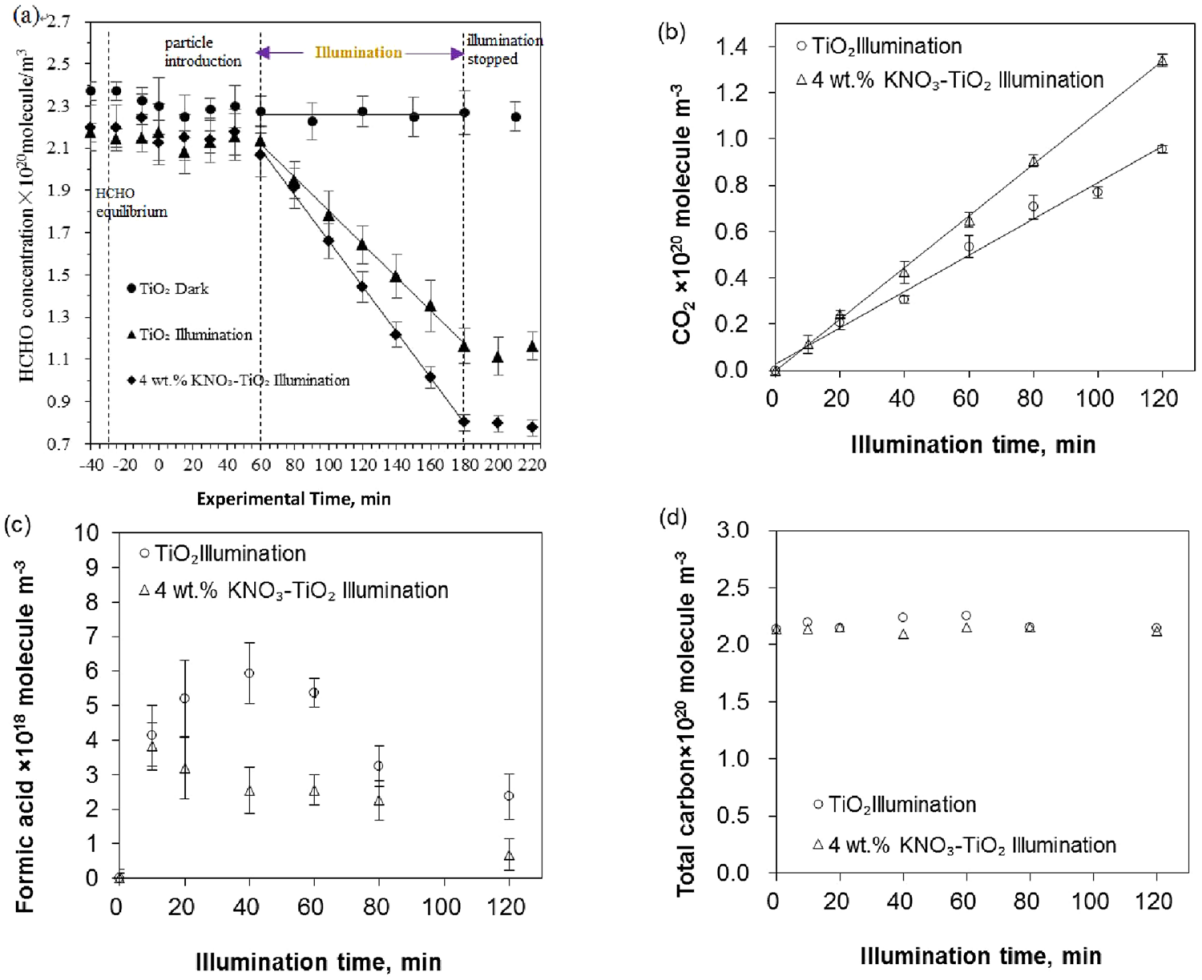
Kinetic curves of reactant and products. (**a**) Concentrations of formaldehyde. (**b**) Concentrations of CO_2_. (**c**) Concentrations of formic acid. (**d**) Concentrations of total carbon. “TiO_2_ dark” is TiO_2_ aerosol in dark condition, “TiO_2_ Illumination” is TiO_2_ aerosol in illuminated condition, “4 wt.% KNO_3_-TiO_2_ illumination” is 4 wt.% KNO_3_ coated TiO_2_ aerosol in illuminated condition. The RH inside the Chamber is 8%.

Firstly, experiments without aerosol introduction were conducted. HCHO was introduced into the chamber under dark conditions without the presence of aerosol particles. After a ~1 hr equilibrium period, the concentration of HCHO was mostly stable and no clear trend was observed (see Supplementary Fig. S1a); when the UV lamps were turned on, a slow decrease of HCHO concentration was observed (see Supplementary Fig. S1b). The time series of HCHO concentration in experiments with TiO_2_ or KNO_3_-TiO_2_ aerosols under both dark and illuminated conditions are shown in Fig. 1a. No decay of HCHO concentration was observed in dark conditions, even when TiO_2_ or KNO_3_-TiO_2_ aerosol was present. A linear decay of HCHO concentration was observed only under illuminated conditions (R^2^ = 0.99). The fitted zero-order reaction rate constant of HCHO on TiO_2_ and KNO_3_-TiO_2_ aerosols was 7.8 × 10^17^ molecule m^−3 ^min^−1^ and 10.8 × 10^17^ molecule m^−3 ^min^−1^, respectively, after the gas-phase photolysis of HCHO was subtracted. This observation suggests that no significant uptake of HCHO on TiO_2_ or KNO_3_-TiO_2_ aerosols exists under dark conditions, which agrees with previous reports of the minor importance of heterogeneous uptake of HCHO on aerosols in dark conditions^11, 12, 16, 18^. In contrast, this observation also suggests a significant but variable photo-enhanced uptake on both TiO_2_ and KNO_3_-TiO_2_ aerosols, which is even faster than HCHO photolysis loss in the gas phase (1.2 × 10^17^ molecule m^−3 ^min^−1^, as can be deduced from Supplementary Fig. S1b). The enhanced uptake rate is ~6.5 and 9.0 times higher on TiO_2_ and KNO_3_-TiO_2_ aerosols, respectively, relative to the gaseous photolysis of HCHO. The photocatalytic effect of TiO_2_ and the synergistic effect of co-existed nitrate is expected and will be discussed below.

Similar experiments with other aerosols, including SiO_2_, KNO_3_-SiO_2_, (NH_4_)_2_SO_4_-TiO_2_ and K_2_SO_4_-TiO_2_ particles, were also carried out to examine the photocatalytic effect of TiO_2_ and the synergistic effect of the co-existed nitrate. Table 1 shows the rate constants on these particles with photolysis rate constant being subtracted. SiO_2_ aerosols presented slight uptake of HCHO, due to some reactive sites existing on the surface of SiO_2_. The comparison between SiO_2_ and TiO_2_ aerosols highlighted the photocatalytic effect of TiO_2_. KNO_3_-SiO_2_ particle showed nearly no uptake of HCHO, maybe because of the blocking effect of KNO3. This implies that the synergistic effect of nitrate on HCHO photo-degradation could only occur when TiO_2_ and nitrate anions are present simultaneously. The photodegradation of HCHO on 4 wt.% K_2_SO_4_-TiO_2_ aerosols was observed to be smaller than on pure TiO_2_ particles, suggesting that K_2_SO_4_ might block reactive sites on TiO_2_ aerosols, and hinder the uptake of HCHO.

**Table 1:** Photoreaction rate constants of HCHO on different aerosol particles.

Particles	Photoreaction rate constant,
	10^17^ molecule m^−3^ min^−1^
SiO_2_	1.2
4 wt.% KNO_3_-SiO_2_	0.2
TiO_2_	7.8
4 wt.% (NH_4_)_2_SO_4_-TiO_2_	2.5
4 wt.% K_2_SO_4_-TiO_2_	6.8
4 wt.% NH_4_NO_3_-TiO_2_	9.0
4 wt.% KNO_3_-TiO_2_	10.8

**Figure 2:**
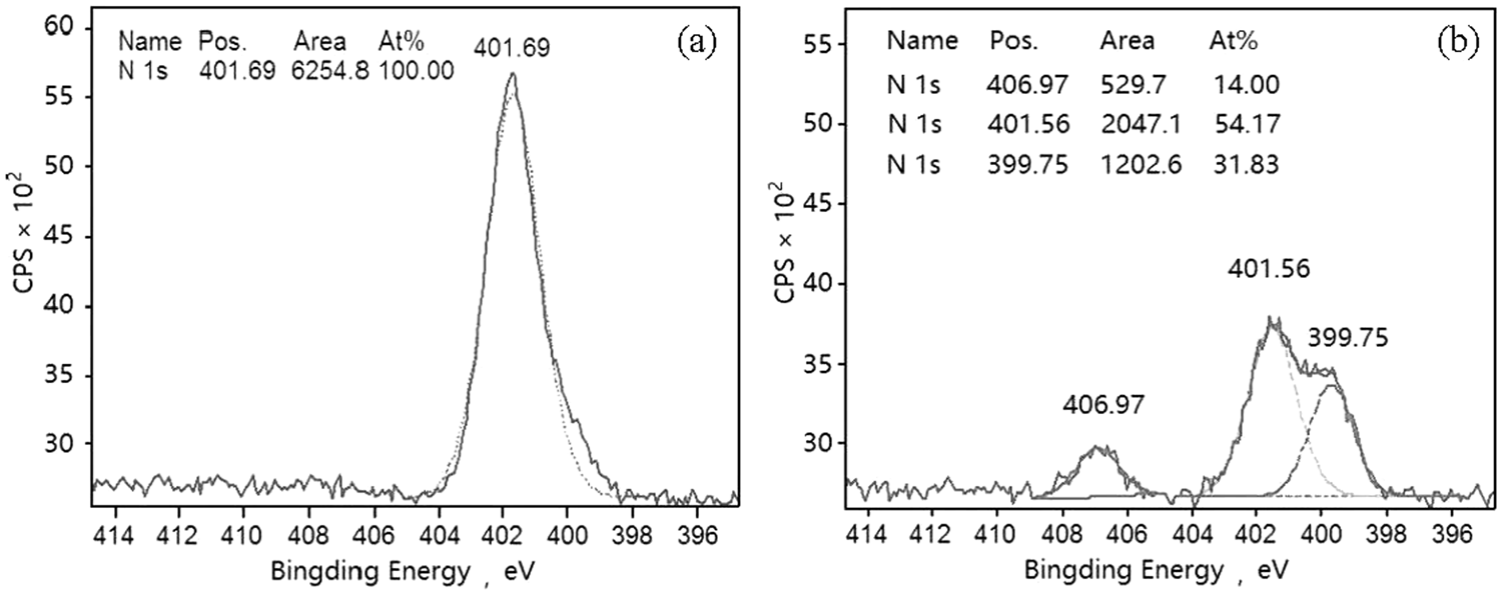
XPS N1s spectra of 20 wt.% (NH_4_)_2_SO_4_-TiO_2_. (**a**) Before photoreaction with formaldehyde. (**b**) After the photoreaction with formaldehyde.

The photolysis rate constant (1.2 × 10^17^ molecule m^−3^ min^−1^) has been subtracted.

(NH_4_)_2_SO_4_-TiO_2_ particles have a much stronger inhibiting effect compared to K_2_SO_4_-TiO_2_ on the photodegradation of HCHO. The significant inhibiting effect of (NH_4_)_2_SO_4_ might be ascribed to NH_4_^+^. Previous studies found that the photogenerated holes, generated by the excitation of TiO_2_, can react with adsorbed H_2_O to produce hydroxyl radicals^38^, which would oxidize negative trivalent N to higher valence nitrogen species, such as NO_2_^−^ or NO_3_^−^^39, 40^. In order to verify this photocatalytic oxidation of NH_4_^+^, XPS N1s spectra of 20 wt.% (NH_4_)_2_SO_4_-TiO_2_ aerosol sample before and after photoreaction with HCHO were measured (Fig. 2). The XPS N1s spectrum of the fresh (NH_4_)_2_SO_4_-TiO_2_ sample presented a clear peak around 401.69 eV, which could be defined as NH_4_^+^^41^. After the (NH_4_)_2_SO_4_-TiO_2_ particles reacted with HCHO under ultraviolet irradiation, the NH_4_^+^ peak was reduced and there were two new peaks emerging at 399.75 eV defined as N^−^^42, 43^ and 406.97 eV defined as NO_3_^−^^44^. The results directly illustrate that the ammonium ion could be oxidized to NO_3_^−^ by the photogenerated holes so as to compete with HCHO photooxidation, thereby having an inhibiting effect on the photodegradation of HCHO. This can also be confirmed by the lower decay rate of HCHO on NH_4_NO_3_-TiO_2_ composite aerosols compared to that on KNO_3_-TiO_2_ aerosols (Table 1).

Figure 1b–d shows the time series of CO_2_, gaseous formic acid, and the total carbon in the illumination experiments along with the photo-enhanced decay of HCHO. The total carbon involving HCHO, CO_2_ and formic acid was near stable throughout the experiments, implying that the mass balance of carbon was almost closed, and that CO_2_ and formic acid were the major products of HCHO photochemical oxidation on aerosols in our experiments (Fig. 1d). CO_2_ appeared to be the main product, as the formation of CO_2_ was much faster than that of formic acid and increased with the photo-enhanced decay of HCHO throughout the illumination experiments (Fig. 1b). The formation rate of CO_2_ in experiments with KNO_3_-TiO_2_ or TiO_2_ aerosols is 1.1 × 10^18^ molecule · m^−3^ · min^−1^ and 7.8 × 10^17^ molecule · m^−3^·min^−1^, respectively. These rates were nearly the same as the HCHO decay rates in these experiments. Gaseous formic acid appeared to be an intermediate, as the formation of formic acid on TiO_2_ aerosols reached its maximum value at about 40 min illumination time and then decreased afterwards, while the formation of formic acid on KNO_3_-TiO_2_ aerosols showed a more complex trend (Fig. 1c). The production of formic acid in the ‘KNO_3_-TiO_2_’ experiment was lower than that in the ‘TiO_2_’ experiment, in contrast to the production of CO_2_. This infers a fast formic acid to CO_2_ conversion in the presence of nitrate in TiO_2_ aerosols, which may be attributed to the synergistic effect of co-existed nitrate.

Overall, both the TiO_2_ particle and the co-existed nitrate promoted the photochemical oxidation of HCHO at a rate dramatically faster than just the HCHO gas phase photolysis in our experiments, with CO_2_ as the main product and formic acid as an intermediate. TiO_2_ is the necessary substance for enhanced HCHO oxidation on the surface, and the synergistic effect relies on the co-existence of nitrate and TiO_2_.

### Rate Dependency on Nitrate Content and RH

**Figure 3:**
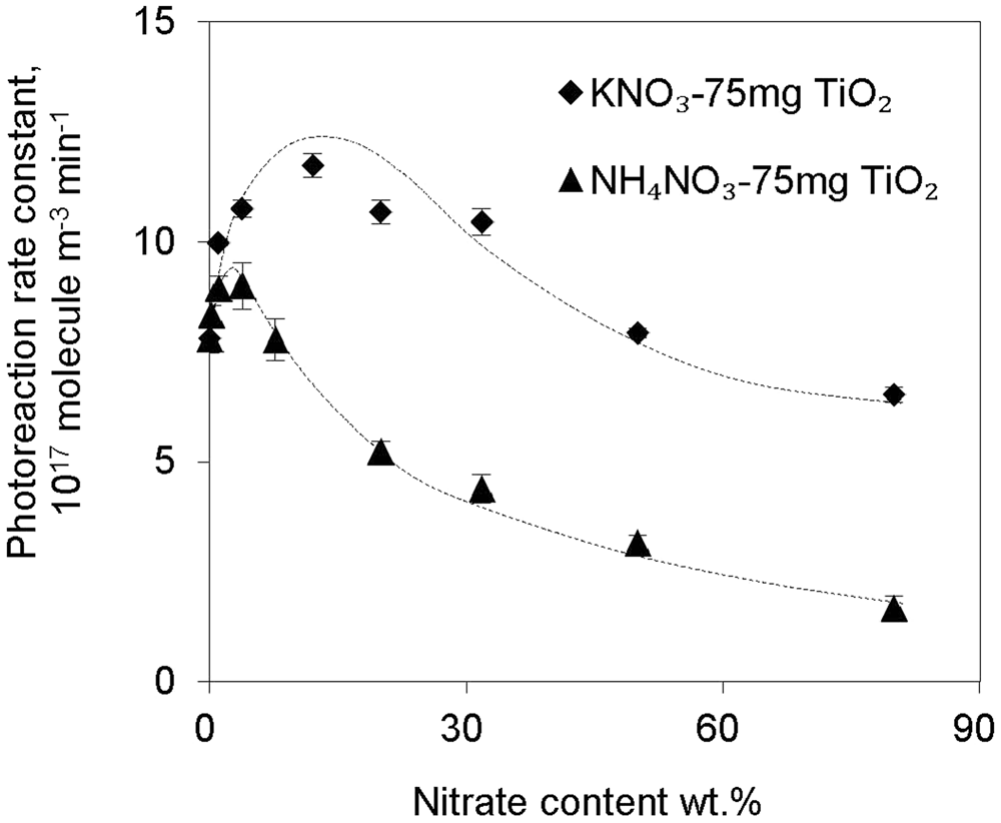
Variations of photoreaction rate constants with nitrate content. Formaldehyde photoreaction rate constants on KNO_3_-TiO_2_ or NH_4_NO_3_-TiO_2_ aerosol as a function of composited nitrate content under 8% RH.

**Figure 4:**
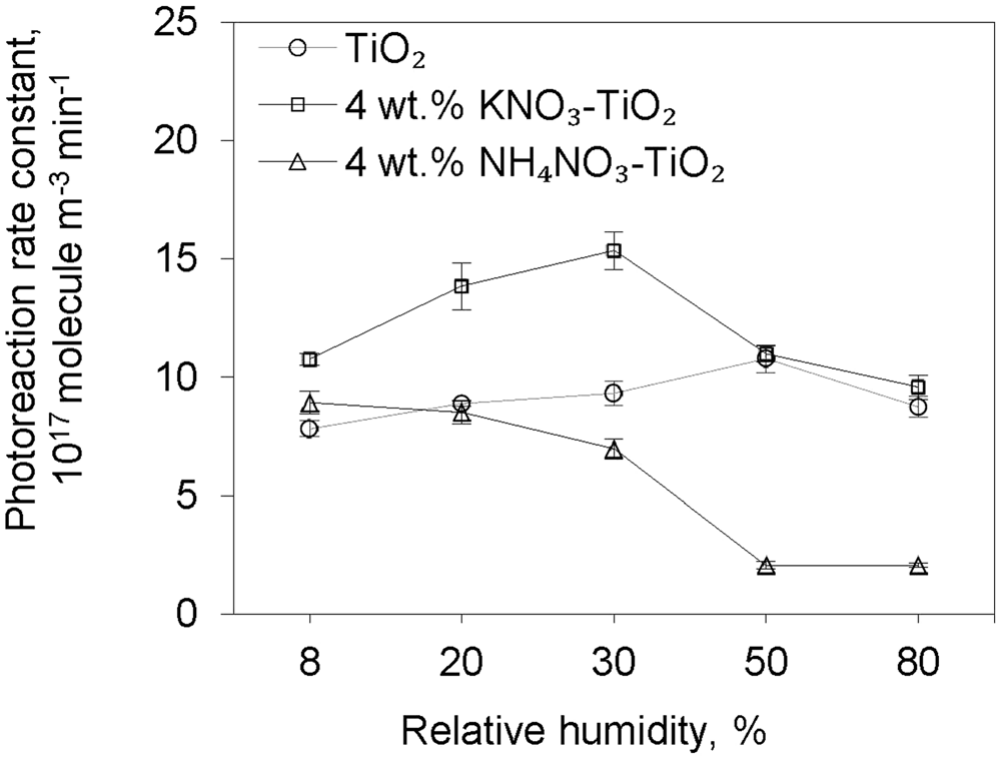
Variations of photoreaction rate constants with relative humidity. Formaldehyde photoreaction rate constants on TiO_2_, 4 wt.% KNO_3_-TiO_2_ or 4 wt.% NH_4_NO_3_-TiO_2_ aerosol as a function of relative humidity.

As TiO_2_ was coated by secondary nitrate at various RH in ambient conditions, the dependency of the heterogeneous oxidation rate of HCHO on nitrate content and relative humidity was key to extrapolating any laboratory results to a real environment. KNO_3_-TiO_2_ and NH_4_NO_3_-TiO_2_ aerosols with different nitrate mass fractions were used in the illumination experiments; the dependence of the oxidation rate constant of HCHO on nitrate content is shown in Fig. 3. The reaction rate constants first increased with nitrate mass fraction at low nitrate loadings, and then decreased with nitrate fraction if nitrate loadings were higher than 12 wt.% for KNO_3_-TiO_2_ or 4 wt.% for NH_4_NO_3_-TiO_2_. As the nitrate content is above 50 wt.% for KNO_3_-TiO_2_ and 8 wt.% for NH_4_NO_3_-TiO_2_, the nitrate-TiO_2_ aerosols demonstrate no stronger reactivity than pure TiO_2_ aerosol. Possible reasons will be discussed below. Similarly, a saddle-shaped dependence of oxidation rate constants of HCHO on RH was observed in the experiments with KNO_3_-TiO_2_ and TiO_2_ aerosols (Fig. 4). The reaction rate constant first increased with RH and then decreased with an optimal value at 30% and 50% for KNO_3_-TiO_2_ and TiO_2_ aerosols, respectively. In the low RH region, KNO_3_-TiO_2_ aerosols presented higher reactivity towards HCHO than pure TiO_2_ aerosols, while at a high RH beyond 50%, the reaction rate constants on the two type of aerosols were similar. On NH_4_NO_3_-TiO_2_ aerosols, the oxidation rate constant of HCHO deceases with RH in the range of 8–80%, and is lower than that on pure TiO_2_ aerosols at RH beyond 20%.

The dependence of the oxidation rate constants of HCHO on nitrate contents and RH shed some light on the underlying mechanism of HCHO oxidation on the aerosols. Previous studies have found that the photogenerated holes on excited TiO_2_ would react with surface H_2_O to produce hydroxyl radicals^38–40^, which is the oxidant for HCHO oxidation. Therefore, H_2_O adsorbed on TiO_2_ surface is essential for this HCHO oxidation reaction. Similarly, NO_3_ radicals are produced from complexed nitrate on illuminated TiO_2_ aerosol, and the NO_3_ radical also behaves as an oxidant, providing an additional driving force for HCHO oxidation on the aerosol (see below). Therefore, the competitive adsorptions of H_2_O with HCHO, nitrate with HCHO ought to be important in determining the oxidation rate of HCHO in this study. On the one hand, the competitive adsorption of nitrate with HCHO on photogenerated holes of TiO_2_ particles might explain the nitrate content dependence (Fig. 3). As the nitrate is accumulatively coated on TiO_2_ aerosols, it blocks the reactive sites and reduces the reaction probability of HCHO with oxidants of both origins. The different dependent patterns of KNO_3_-TiO_2_ aerosols and NH_4_NO_3_-TiO_2_ aerosols in Fig. 3 might be due to another pair of competitive reactions, i.e., reactions of oxidants with HCHO and NH_4_^+^, which is also reflected by previous analyses of Fig. 2. On the other hand, the competitive adsorption of H_2_O with HCHO might explain the RH dependence for the case of KNO_3_-TiO_2_ and TiO_2_ aerosols (Fig. 4). An optimal amount of H_2_O on the aerosols facilitates the decomposition of H_2_O by photogenerated holes on excited TiO_2_, thus the formation of ·OH oxidants. Conversely, excessive adsorbed water, like excessive nitrate, affects the reaction by inhibiting the contact between HCHO and the oxidants^45^, or changing the oxide coordinated nitrate to water solvated nitrate, thus reducing the reaction rate^46^. The different hydroscopic properties of nitrate salts make the dependence more complex. For example, the KNO_3_ in KNO_3_-TiO_2_ particles helps to adsorb moisture, leading to a greater number of OH radicals being generated at low surface water content, but more active sites being blocked by the nitrate solution layer on aerosols under high surface water content, relative to pure TiO_2_ aerosols. The Langmuir-Hinshelwood type of dependence of the reaction rate on RH is not observed in the ‘NH_4_NO_3_-TiO_2_’ experiment. NH_4_NO_3_ salt has low deliquescence point and adsorbed water may make NH_4_^+^ easily dissolved. This would make the reaction between NH_4_^+^ and OH radical (as discussed before) easier than the reaction between HCHO and OH radicals. So the adsorbed water showed negative effect on HCHO photodegradation over NH_4_NO_3_-TiO_2_. Another possible reason for no peak of RH is the inability to control RH below 8% in our environmental chamber.

## Discussion

Based on the data above and some references, a mechanism for the oxidation of HCHO on TiO_2_ particles under illuminated conditions can be proposed. As shown in Supplementary Eqs S1–S13 ^11, 18, 47–50^. When TiO_2_ was irradiated with light energy higher than its bandgap (that is, with wavelengths lower than 387 nm), electron-hole pairs generated on the surface could react with H_2_O and O_2_, producing reactive oxygen species (ROS) (such as ·OH, HO_2_· and H_2_O_2_) (see Supplementary Eqs S1–S5), as suggested elsewhere^18, 49^. The ROS, especially OH radicals, played important roles in oxidizing HCHO. *Xu et al*. detected H_2_COO as the transitional product (shown in Supplementary Eqs S6–S9)^11^. HCHO can also directly react with HO_2_· to generate HCOOH^48^ (see Supplementary Eq. S10). The formic acid/formate could further be oxidized to CO_2_ by OH (see Supplementary Eqs S11–S13). In addition, the photogenerated holes could also react directly with adsorbed HCHO and produce CO_2_^47^. Competition reactions between HCHO and other molecules have not been shown.

**Figure 5:**
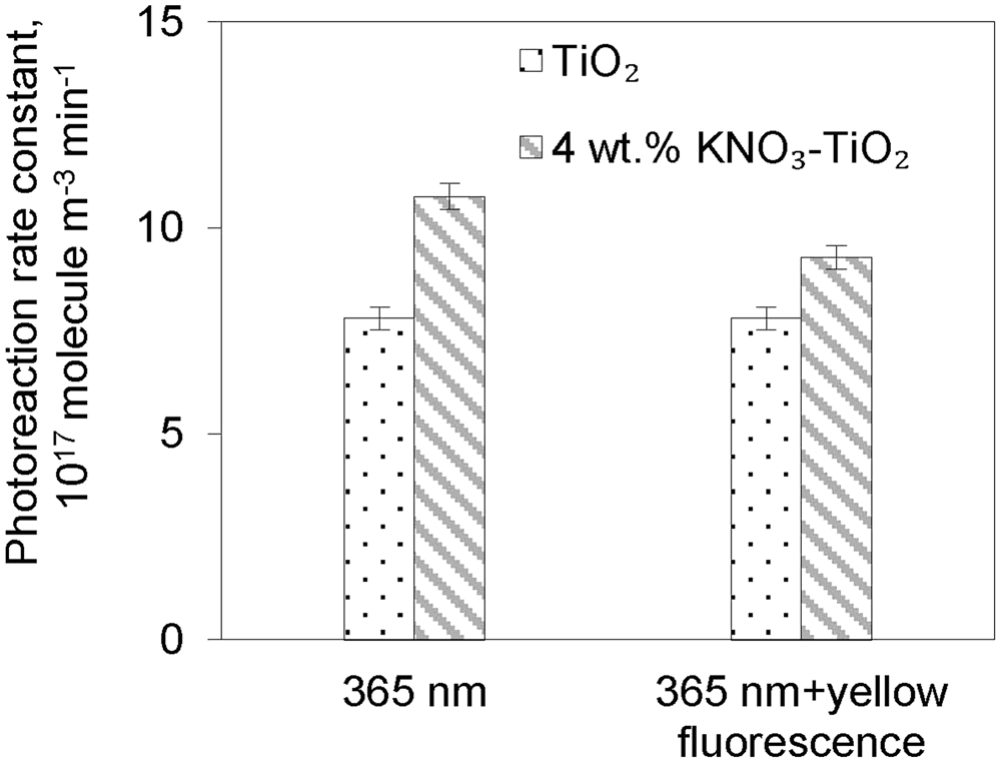
Photoreaction rate constants with light illumination. Formaldehyde photoreaction rate constants on TiO_2_ or 4 wt.% KNO_3_-TiO_2_ aerosol in the condition of 8% RH under light illumination of “365 nm” or “365 nm + yellow fluorescence”, respectively.

As illustrated in Fig. 1, a substantial enhancement in HCHO uptake on aerosol was observed in the presence of nitrate salts. This might be attributed to the electron-trapping effect of nitrate anion, which limited charge-carrier recombination at the TiO_2_ surface and thus increased the availability of holes for oxidative process of adsorbed HCHO^51^. NO_3_^−^ could also react directly with photogenerated holes to generated NO_3_ radicals (Eqs 1 and 2), according to previous investigations of the photochemistry of illuminated nitrate-TiO_2_ particles^52^. NO_3_ radicals generated on the surface could effectively oxidize HCHO, with CHO· and HNO_3_ as the products (Eq. 3)^53^. CHO· further participated in a series of reactions to finally generate CO_2_ (Eqs 6 and 7). Meanwhile, NO_3_ radicals underwent a rapid photolysis under visible light irradiation to generate NO and NO_2_ through Eqs (4) and (5), with Eq. (4) as the prominent pathway^54, 55^. This gave us an opportunity to verify the existence and effect of NO_3_ radicals in our experiment system. Figure 5 shows the photoreaction rate constants of HCHO on TiO_2_ and KNO_3_-TiO_2_ aerosols under “365 nm lamp” illumination and under both “365 nm lamp” and yellow fluorescence lamp (450–750 nm) illumination. The oxidation rate constants of HCHO on TiO_2_ aerosol were comparable under these two illumination conditions. The probable reason for this is that TiO_2_ is not sensitive to visible light. However, the rate constant on KNO_3_-TiO_2_ aerosol under illumination of both lamps was lower than that under only the “365 nm lamp”, indicating a reduced oxidation rate due to NO_3_ radical photolysis by visible light. The observation of the rate decrease of HCHO due to NO_3_ photolysis provides experimental evidence for the existence of NO_3_ for the first time.

1 $${{\rm{TiO}}}_{2}+hv\,({\rm{\lambda }} < 390\,{\rm{nm}})\to {{\rm{e}}}^{-}+{{\rm{h}}}^{+}$$

2 $${{\rm{NO}}}_{3}^{-}+{{\rm{h}}}^{+}\to {{\rm{NO}}}_{3}\cdot $$

3 $${{\rm{NO}}}_{3}\cdot +{\rm{HCHO}}\to {\rm{CHO}}\cdot +{{\rm{HNO}}}_{3}$$

4 $${{\rm{NO}}}_{3}\cdot +hv\,({\rm{\lambda }}\le 640\,{\rm{nm}})\to {{\rm{NO}}}_{2}+{\rm{O}}({}^{3}{\rm{P}})$$

5 $${{\rm{NO}}}_{3}\cdot +hv\,(585{\rm{nm}}\le {\rm{\lambda }}\le 640\,{\rm{nm}})\to {\rm{NO}}+{{\rm{O}}}_{2}$$

6 $${\rm{CHO}}\cdot +{{\rm{O}}}_{2}\to {\rm{CH}}({\rm{O}}){{\rm{O}}}_{2}\cdot $$

7 $${\rm{CH}}({\rm{O}}){{\rm{O}}}_{2}\cdot +{\rm{NO}}+{{\rm{O}}}_{2}\to {{\rm{NO}}}_{2}+{{\rm{HO}}}_{2}\cdot +{{\rm{CO}}}_{2}$$

Taking into account that both OH and NO_3_ radicals are coming from photogenerated holes reacted with adsorbed H_2_O and nitrate, respectively, there would exist a competition of the two processes. The mass effect of TiO_2_ on the reaction rate of HCHO is depicted in Supplementary Fig. S2. The reaction rate kept increasing with added mass of TiO_2_ (without any nitrate doping) below 60 mg (see Supplementary Fig. S2(a)). After that, the reaction rate levelled off and reached a plateau. Below this threshold of 60 mg, the almost linear trend is due to the increase of photogenerated holes and therefore of OH radicals (through the following reaction h^+^  + H_2_O → HO· + H^+^), responsible for the oxidation of HCHO. At higher TiO_2_ loadings, the plateau is due to radical self-reactions and various recombination processes. These observations nicely fit standard trends in TiO_2_ based photocatalysis, even at the lower mass investigated here, clearly indicating that it controls the degradation of HCHO. While photocatalysis dominates the degradation of HCHO, the exact pathway in presence of nitrate depends on its actual mass, as shown in Supplementary Fig. S2(b). At low TiO_2_ masses, the nitrate anions react with available holes (NO_3_^−^ + h^+^_vb_ → NO_3_·) and reduce the amount of generated OH radicals, overall this leads to a reduction of the observed reaction rate (nitrate radicals being less reactive than OH radicals). At different loadings, however the situation is quite different as a clear enhancement can be observed when significant amount of NO_3_ can be produced. Thus nitrate presents positive effect on the photodegradation of HCHO for KNO_3_-75 mgTiO_2_ sample. Therefore, there is a competition of photogenerated holes to produce OH and NO_3_ radicals, which are both responsible for the oxidation of HCHO, with OH radical as the major one. This is another evidence that NO_3_ radicals are produced in the system and can present its synergistic effect with appropriate amount of TiO_2_ loading.

Enhanced oxidative decays of HCHO on both TiO_2_ aerosols and nitrate-TiO_2_ aerosols were observed under illuminated conditions. The photocatalytic effect of TiO_2_ plays a key role in the enhanced oxidation of HCHO. The synergistic effect of nitrate is ascribed to the generation of NO_3_ radicals on excited TiO_2_ aerosols. Currently, it is out of reach to measure *in-situ* the surface concentration of nitrate radicals with state-of-the-art analytical tools. However, we have experimentally proved the presence of NO_3_ radicals, as discussed above. NH_4_^+^ reacts competitively with photogenerated holes on the TiO_2_ aerosol, and produces high valence nitrogen species. The Langmuir-Hinshelwood type of dependence of the oxidation rate of HCHO on nitrate and water content highlights the competitive adsorption and reaction of the reactants, i.e. HCHO with nitrate, HCHO with H_2_O, on the reactive sites of TiO_2_ aerosol.

The oxidation rate of HCHO on both TiO_2_ aerosol and nitrate-TiO_2_ aerosol is around one order of magnitude faster than the photolysis of HCHO in the gas phase. The uptake coefficient is calculated to be in the range of 0.43–1.68 × 10^−7^ under our experimental conditions (see Supplementary: Calculation of uptake coefficient and Table S1), which is ~1–2 orders of magnitude higher than previous reports^11, 12, 16^, implying a novel HCHO sink of potential atmospheric significance. The photocatalytic oxidation rate of HCHO might linearly depend on TiO_2_ mass fraction in dust aerosol in ambient conditions, which is much lower than the TiO_2_ mass fraction used in our experiments. Assuming a TiO_2_ mass fraction of 5% in mineral dust and a comparable surface density of mineral dust aerosol to our experimental value (2 × 10^4 ^μm^2^ cm^−3^), the heterogeneous oxidation decay of HCHO on dust aerosol is at least comparable to its photolysis in the gas phase in typical dust events^20^. Furthermore, the enhanced uptake of HCHO on low NO_3_^−^ loading TiO_2_ aerosol, as indicated in our study, suggests the importance of this kind of reaction in dust storm episodes when the mass percentage of NO_3_^−^ is less than 40%^56^. On non-dust days, the heterogeneous oxidation decay of HCHO is less important, but cannot be neglected in HCHO budget analysis. Finally, our results demonstrate the need for more experiments employing ambient dust aerosols of various atmospheric aging processes under various environmental conditions, to parameterize the oxidative decay of HCHO on mineral dust better.

## Methods

### Environmental Chamber

An environmental chamber was built for studying the heterogeneous reaction of HCHO with TiO_2_ and nitrate-doped TiO_2_ aerosols (see Supplementary Fig. S3). The main body of the chamber is a 400 L FEP bag (Mitsubishi, Japan, designed by Safelab company, Beijing) having a pillow shape after inflation with a size of 1.15 m (length) × 1.40 m (height) × 0.55 m (width). It is vertically hung on an aluminum frame and is covered with a light-shading black cloth.

The chamber is capable of temperature, light intensity and relative humidity control, and is equipped with contamination-free carrier gas supply and a particle introduction system. Temperature was controlled to be around 295 K in the laboratory by air conditions. One light source with a solar-mimicking UV spectrum of 320–400 nm was used, with the main wavelength of 365 nm, being called the 365 nm lamp hereafter. In some experiments, one yellow fluorescence lamp in the wavelength range from 450–750 nm was also used for comparison, being called the yellow fluorescence lamp hereafter. The output spectra of both light sources are presented in Supplementary Fig. S4. Zero air was obtained from compressed air after purification with activated carbon, filtering aerosol (Jiechi purification equipment Limited, Shanghai) and drying with porous silicon. Saturated water vapor was obtained via a water bubbler, and introduced into the chamber together with dry air at certain flow rates to control the desired relative humidity. Aerosol was introduced through the particle introduction system. This consists of a high-pressure pipe and particle holder tube. Sample powder was first placed inside the holder tube and then sprayed into the chamber by a transient high-pressure high pure nitrogen gas (99.9999%, Huayuan Gas Company, Beijing).

The chamber is also equipped with various inlets, outlets and detection instruments. The particle size distribution was measured by a Scanning Nano Particle Spectrometer (SNPS-n20, HCT, Korea, Sheath air flow: Sample air flow = 10:1). Other outflow from the chamber was first filtered prior to any further analysis to protect the instruments from contamination with particles. Reactant HCHO was generated by thermolysis of paraformaldehyde at 70 °C and was brought into the chamber by the carrier gas at a flow rate of 100 mL/min and detected via acetyl acetone spectrophotometric method using a UV-Vis spectrophotometer (T6, PERSEE, Beijing). CO_2_ was detected by gas chromatography (SP3429, FID detector, Molecular sieve column, Column temperature, 100 °C, Injection temperature, 100 °C) equipped with an oxidation furnace. Gaseous formic acid was first scrubbed in a porous-glass-plate absorption tube by deionized water and then detected by ion chromatography (US Dionex, ICS-900, AS-14 anion column with AG-14 protection column, ASRS-4 mm anion suppressor, ECD-1 detector) with 1.25 mM sodium borate solution as eluent.

### Particulate Sample Preparation and Chemicals

In our experiments, two nitrate salts, potassium nitrate or ammonium nitrate, were complexed with TiO_2_ aerosols to evaluate the nitrate effect on HCHO uptake and possible influences of the cations. First, a series of nitrate solutions of different concentrations was prepared. Then, 300 mg of TiO_2_ particles were mixed in 1 mL nitrate solution to obtain a mash. The mash was dried in an oven and then ground carefully to ensure a uniform composite of particles. Since ammonium nitrate decomposes at 110 °C, the drying temperature was set to 90 °C. The nitrate content in the composite aerosol samples ranged from 0.2–80 wt.%. For the purpose of comparison, 4 wt.% (NH_4_)_2_SO_4_-TiO_2_, 20 wt.% (NH_4_)_2_SO_4_-TiO_2_, 4 wt.% K_2_SO_4_-TiO_2_ and 4 wt.% KNO_3_-SiO_2_ samples were also prepared. The blank TiO_2_ or SiO_2_ samples were solved in pure water with the same procedure as mentioned above.

The chemicals used are commercial products. Titanium dioxide is Degussa P25 from Germany (50 nm, 54 m^2^/g), SiO_2_, KNO_3_, NH_4_NO_3_, (NH_4_)_2_SO_4_ and K_2_SO_4_ are all analytical reagents (>96%).

### Experiments of Formaldehyde with Particles

Before each experiment, the chamber was cleaned by pumping and inflating with zero air for two cycles. The deflated chamber was inflated by 200 L zero air, following by the introduction of HCHO, and then the chamber was filled up with zero air to about 400 L. The concentrations of HCHO, CO and CO_2_ were detected as a function of time. It took about 60 min for the HCHO concentration to be stable. After the HCHO concentration became stable, particulate samples were introduced, and the particle size distribution of aerosol inside the chamber was also monitored as a function of time. The concentration of HCHO decreased once particles were introduced, and it took another 30 min to reach stable plateau again. Meanwhile, large aerosol particles tended to settle down on the inner wall of the chamber within 30 min of the introduction; thereafter a relative stable particle number density of about 4000 cm^−3^ in a mono-modal distribution with a peak particle size around 100 nm was observed (see Supplementary Fig. S5). Compared to an ambient dust aerosol observation in Beijing, our laboratory-generated TiO_2_ aerosol contains a comparable number of particles beyond 40 nm, but fewer particles smaller than 40 nm^57^. The surface density of aerosols during the whole experiment stage was stable at around 2 × 10^4^ μm^2^ cm^−3^, which was comparable to that observed in dust events in Beijing, 1–2 orders of magnitude higher than observations in the urban area, and up to three orders of magnitude higher than observations in the background area^8, 57^. After the concentrations of both HCHO and aerosol became stable, the lamps were turned on and the concentrations of HCHO, CO, CO_2_ and gaseous formic acid were monitored.

The blank experiment without aerosol (Supplementary Fig. S1) shows that the photolysis frequency of HCHO in the gas phase fitted to be around 2 × 10^−5^ s^−1^. The photolysis frequency was roughly comparable to the “standard” photolysis frequency of HCHO during typical tropical summer conditions on the ground (solar elevation angle θ = 0°)^58^, suggesting that the light intensity in our experiment was environmentally relevant. In addition, the solar light-mimicking spectrum of our light source in the 300–365 nm region overlaps with the HCHO spectrum in the UV region. Overall, the characteristics of the light source enabling the photochemical reaction are environmentally relevant.

## Electronic supplementary material

Supplementary Information
