# Supplementary material for: Synergistic effect of nitrate-doped TiO2 aerosols on the fast photochemical oxidation of formaldehyde
Source: Sci Rep. 2017 Apr 25;7:1161. doi: 10.1038/s41598-017-01396-x (PMC5430731; doi:10.1038/s41598-017-01396-x)
Supplement: Supplementary file 1 — Supplementary Information [file 41598_2017_1396_MOESM1_ESM.pdf]

Supplementary information for

Synergistic effect of nitrate-doped TiO_2 aerosols on the fast photochemical oxidation of formaldehyde

Jing Shang[1*], Wei Wei Xu[1], Chun Xiang Ye[1], Christian George[2*], and Tong Zhu[1]

[1] State Key Joint Laboratory of Environmental Simulation and Pollution Control, College of Environmental Sciences and Engineering, Peking University, Beijing 100871, People's Republic of China

[2] Université Lyon 1, CNRS, UMR 5256, IRCELYON, Institut de recherches sur la catalyse et l'environnement de Lyon, 2 avenue Albert Einstein, F-69626 Villeurbanne, France

HCHO concentration variations without particles under dark and UV irradiation (Figure S1); TiO_2 mass dependence on photoreaction rate constants of HCHO in case of sole TiO_2 or NO_3-TiO_2 composite (Figure S2). A schematic drawing of the Environmental Chamber (Figure S3); Spectra distribution of light sources (Figure S4); Changes of particles size distribution in the chamber (Figure S5); Photocatalytic reactions mechanism equations (Text S1); Uptake coefficient calculation processes (Text S2) and uptake coefficients values at different RH and nitrate concentrations (Table S1).

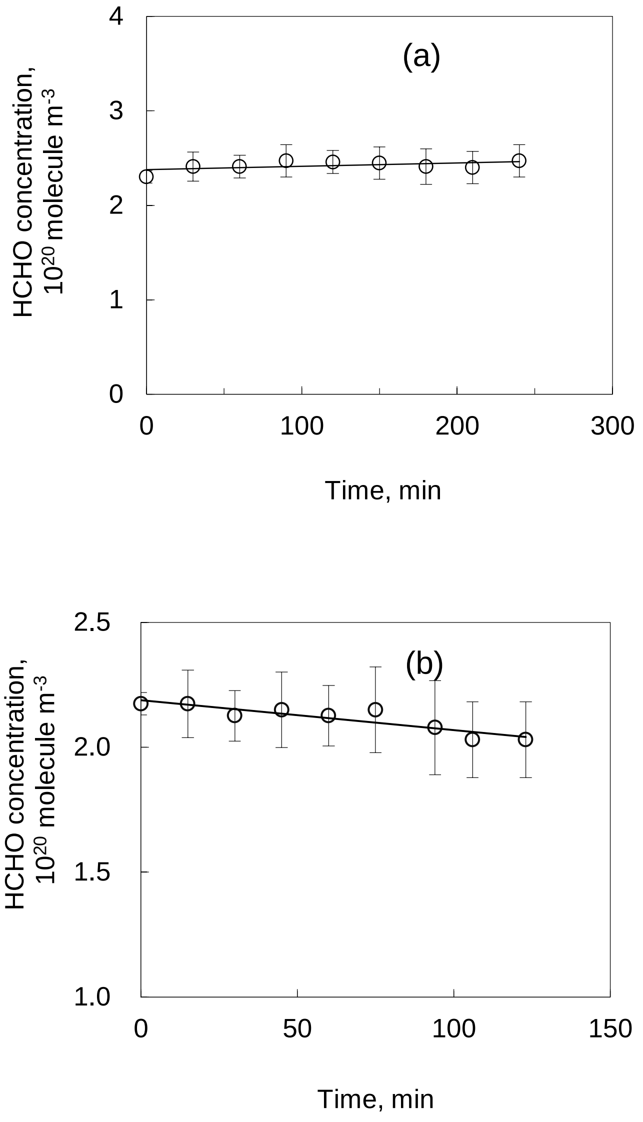

Figure S1. Time series of HCHO concentration in the absence of aerosol particles under dark condition (a) and under 365 nm wavelength UV irradiation (b).

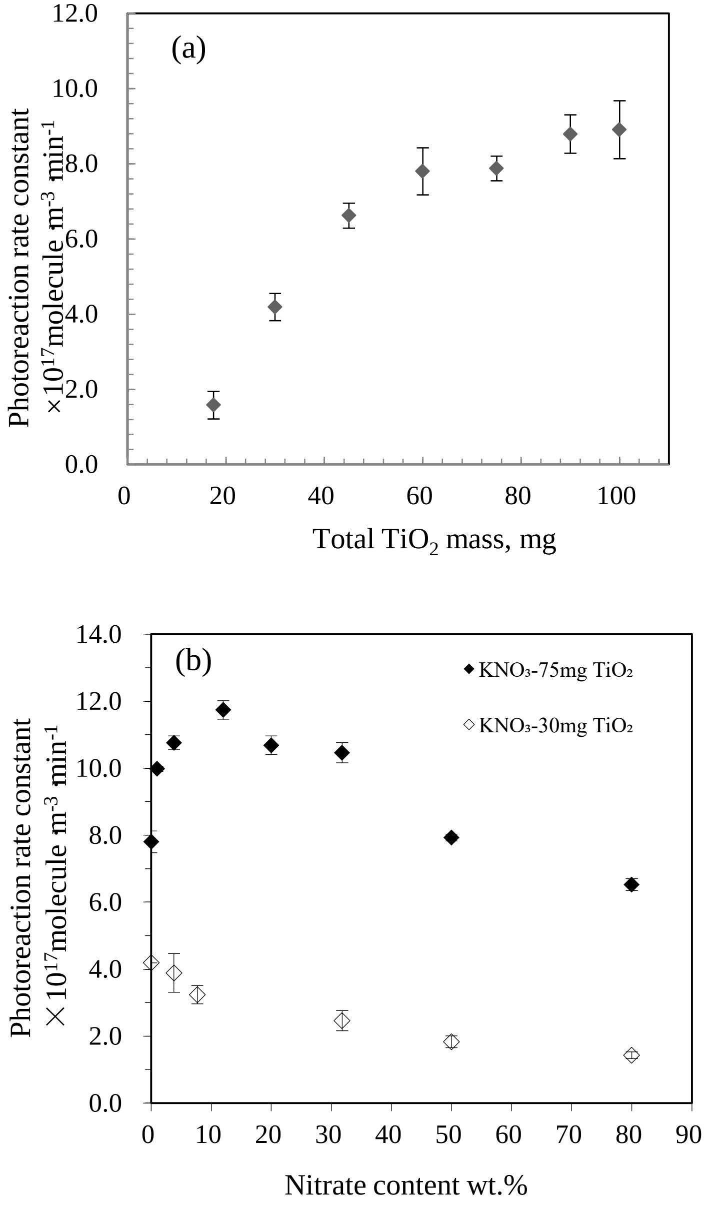

Figure S2. (a): Photoreaction rate constants of HCHO with TiO_2 as function of total mass of TiO_2 in chamber. (b): Photoreaction rate constants of HCHO with KNO_3-75mgTiO_2, KNO_3-30mg TiO_2 particles as function of nitrate content. Experimental conditions: HCHO initial concentration = (2.1-2.4) $\times 10^{20}$ molecule m^{-3}, RH=8%, T=25℃.

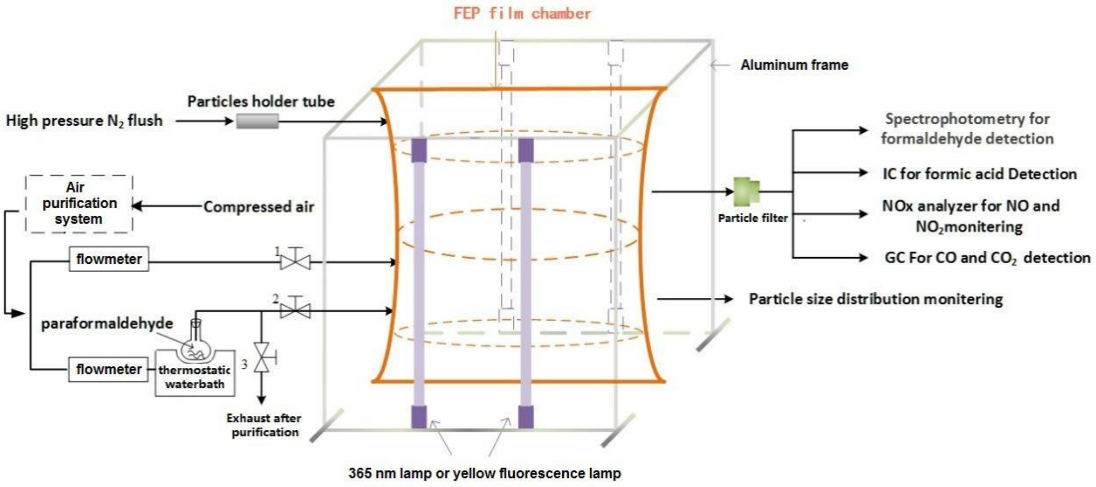

Figure S3. Schematic of FEP film environmental simulation chamber

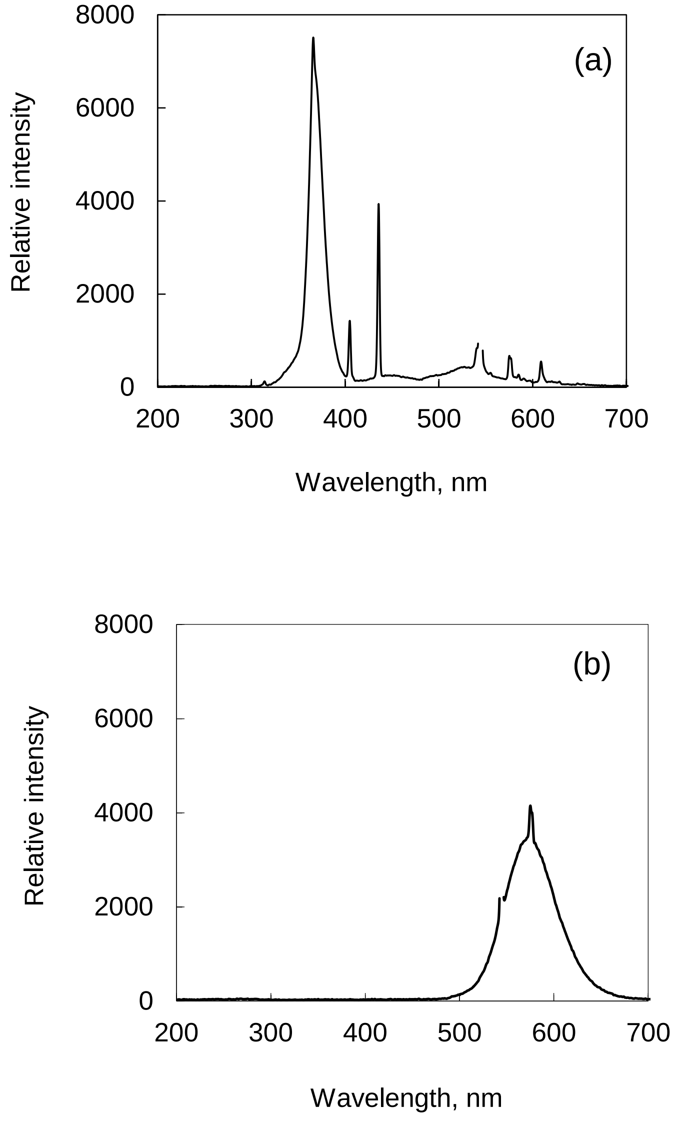

Figure S4. Spectra of 365 nm UV lamp (a) and yellow fluorescence lamp (b).

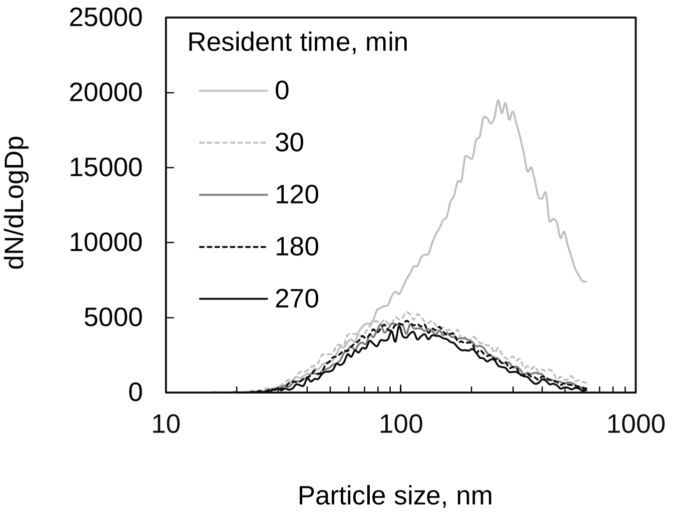

Figure S5. Changes of particles size distribution of TiO_2 aerosol with resident time in the aerosol chamber.

Text S1. Photocatalytic reactions mechanism equations:

$TiO_2 + h\nu$ (λ<390nm) $\rightarrow e^- + h^+$ (S1)

$h^+ + H_2O \rightarrow HO\cdot + H^+$ (S2)

$2OH\cdot \rightarrow H_2O_2$ (S3)

$e^- + O_2 \rightarrow O_2^-$ (S4)

$O_2 + H^+ + e^- \rightarrow HO_2\cdot$ (S5)

$HCHO + OH\cdot \rightarrow H_2COO + H^+$ (S6)

$HCHO + O_2^- \rightarrow H_2COO + O$ (S7)

$H_2COO + HCHO \rightarrow HCOO^- + CH_3O$ (S8)

$HCOO^- + H^+ \rightarrow HCOOH$ (S9)

$HCHO + HO_2\cdot \rightarrow HCOOH + HO\cdot$ (S10)

$HCOO^- + \cdot OH \rightarrow CO_2 + H_2O$ (S11)

$HCOO^- + H^+ \rightarrow H^+ + \cdot CO_2^-$ (S12)

$\cdot CO_2^- + [O]/\cdot OH/H^+ \rightarrow CO_2$ (S13)

Text S2. Calculation of uptake coefficient

The uptake coefficient is defined as the ratio of the number of consumed gas molecules in the reaction with condensed phase and the total number of collision molecules on surface of the condensed phase. For the reaction of formaldehyde and TiO_2, the consumption of formaldehyde molecules on particles can be characterized by the reaction rate of HCHO on particles. The equation of uptake coefficientcan be expressed as:

$$\gamma = \frac{-d[HCHO]/dt}{Z}$$

Z is the total frequency of collision of HCHO on the particles, which is related to the effective contact area of reaction A, formaldehyde molecular concentration [HCHO] and the average velocity of formaldehyde molecules , and can be represented by the following formula:

$$Z = \frac{1}{4}A[HCHO]\bar{c}$$

The BET surface area S_{BET} is used to characterize the effective contact area of the reaction collision. According to reaction kinetics curve, the reaction of formaldehyde with TiO_2 is zero order reaction in our reaction system. The reaction rate constant can be presented as . Then the uptake coefficient can be calculated from the following formula, where Cmass is the total concentration of particles in the reaction chamber:

$$\gamma = \frac{4k}{S_{BET}C_{mass}[HCHO]\bar{c}}$$

The initia concentration of HCHO is controlled about 2.0×10^{20} molecule m^{-3}. The uptake coefficients at different humidity and different nitrate concentration on TiO_2 and KNO_3-TiO_2 particles are reported below (Table S1).

Table S1. Summary of uptake coefficient values of HCHO on TiO_2 and KNO_3-TiO_2 particles

Parameters	nitrate concentration (%)	RH (%)	Particles mass (mg)	(10^{17} molecule $m^{-3} \cdot$ min^{-1})	γ ($\times 10^{-7}$)
Different RH	0	8	75	7.80	0.79
		20		8.88	0.90
		30		9.33	0.94
		50		10.79	1.09
		80		8.75	0.88
Different RH	4	8	78.2	10.76	1.17
		20		13.85	1.52
		30		15.35	1.68
		50		10.99	1.20
		80		9.58	1.05
Different nitrate concentration in KNO_3-TiO_2	0	8	75	7.80	0.79
	1		75.8	9.98	1.06
	4		78.2	10.76	1.17
	12		85.25	11.74	1.35
	20		93.7	10.69	1.30
	32		110.25	10.46	1.40
	50		150	7.93	1.17
	80		375	6.52	0.43
